# Risk Prediction for Locoregional Recurrence in Epidermal Growth Factor Receptor-Mutant Stage III-pN2 Lung Adenocarcinoma after Complete Resection: A Multi-center Retrospective Study

**DOI:** 10.7150/jca.47119

**Published:** 2020-08-25

**Authors:** Qi-Wen Li, Bo Qiu, Wen-Hua Liang, Jun-Ye Wang, Wan-Ming Hu, Tian Zhang, Shuang-Bing Xu, José López, Nai-Bin Chen, Min-Zhang Guo, Yi Zhao, Ling-Juan Chen, Song-Ran Liu, Jing-Ping Yun, Jin-Yu Guo, Si-Yu Wang, Xin Wang, Li Zhang, Dong-Sheng Yue, Zhong-Xing Liao, Steven H. Lin, Hao Long, Qing-Song Pang, Hui Liu

**Affiliations:** 1 Department of Radiation Oncology, State Key Laboratory of Oncology in South China, Collaborative Innovation Center for Cancer Medicine, Sun Yat-sen University Cancer Center, Guangzhou, China; 2 Lung Cancer Research Center, Sun Yat-sen University, Guangzhou, China; 3 Department of Thoracic Surgery and Oncology, The First Affiliated Hospital of Guangzhou Medical University/State Key Laboratory of Respiratory Disease/National Clinical Research Center of Respiratory Disease, Guangzhou, China; 4 Department of Thoracic Surgery, State Key Laboratory of Oncology in South China, Collaborative Innovation Center for Cancer Medicine, Sun Yat-sen University Cancer Center, Guangzhou, China; 5 Department of Pathology, State Key Laboratory of Oncology in South China, Collaborative Innovation Center for Cancer Medicine, Sun Yat-sen University Cancer Center, Guangzhou, China; 6 Department of Radiation Oncology, Tianjin Medical University Cancer Institute and Hospital, Tianjin, China; 7 Union Hospital Cancer Center, Tongji Medical College, Huazhong University of Science and Technology, Wuhan, China; 8 Group of Technological Innovation, Radiation Oncology, University Hospital Virgen del Rocio, Sevilla, Spain; 9 Department of Medical Oncology, State Key Laboratory of Oncology in South China, Collaborative Innovation Center for Cancer Medicine, Sun Yat-sen University Cancer Center, Guangzhou, China; 10 Department of Lung Cancer, Lung Cancer Center, Tianjin Medical University Cancer Institute and Hospital, Tianjin, China; 11 Department of Radiation Oncology, M.D. Anderson Cancer Center, Houston, Texas, USA

**Keywords:** Locoregional recurrence, postoperative radiotherapy, lymph node metastasis, extranodal extension, Robinson classification

## Abstract

**Background:** This study aimed to develop a predictive model based on the risk of locoregional recurrence (LRR) in epidermal growth factor receptor (*EGFR*)-mutant stage III-pN2 lung adenocarcinoma after complete resection.

**Methods:** A total of 11,020 patients with lung surgery were screened to determine completely resected *EGFR*-mutant stage III-pN2 lung adenocarcinoma. Patients were excluded if they received preoperative therapy or postoperative radiation therapy (PORT). The time from surgery to LRR was recorded. Clinicopathological variables with statistical significance predicting LRR in the multivariate Cox regression were incorporated into the competing risk nomogram. Patients were then sub-grouped based on different recurrence risk as a result of the nomogram.

**Results:** Two hundred and eighty-eight patients were enrolled, including 191 (66.3%) with unforeseen N2 (IIIA1-2), 75 (26.0%) with minimal/single station N2 (IIIA3), and 22 (7.6%) with bulky and/or multilevel N2 (IIIA4). The 2-year overall cumulative incidence of LRR was 27.2% (confidence interval [CI], 16.3%-38.0%). IIIA4 disease (hazard ratio, 2.65; CI, 1.15-6.07; *P*=0.022) and extranodal extension (hazard ratio, 3.33; CI, 1.76-6.30; *P*<0.001) were independent risk factors for LRR and were incorporated into the nomogram. Based on the nomogram, patients who did not have any risk factor (low-risk) had a significantly lower predicted 2-year incidence of LRR than those with any of the risk factors (high-risk; 4.6% *vs* 21.9%, *P*<0.001).

**Conclusions:** Pre-treatment bulky/multilevel N2 and pathological extranodal extension are risk factors for locoregional recurrence in *EGFR*-mutant stage III-pN2 lung adenocarcinoma. Intensive adjuvant therapies and active follow-up should be considered in patients with any of the risk factors.

## Introduction

Locoregional recurrence (LRR) has been reported in over 20%-30% of patients with stage IIIA lung adenocarcinoma.^1^-^2^ Postoperative radiation therapy (PORT) has a theoretical potential to improve locoregional control and other long-term outcomes in stage III-pN2 patients, which is also supported by results from large-scale retrospective studies.^3^-^5^ However, stage III-pN2 patients consist of heterogenous subgroups with significantly different LRR risk, thus only 29%-64% of pN2 patients were referred to PORT in clinical practice 3-5. The commonly recognized risk factors include the status of lymph node involvement and certain histological characteristics,^9^ but most of them are experience-based rather than evidence-based. A precise identification of the high-risk patients is important for the recommendation of PORT administration.

Suggested by our previous study, epidermal growth factor receptor (*EFGR*) mutation was associated with a lower risk of LRR in completely resected stage III-pN2 lung adenocarcinoma. Among these patients, only 18.6% of all noted recurrences occurred in the locoregional site, which was significantly lower than those of *EGFR* wild-type counterparts (36.7%).^10^ Besides, recent reports revealed even better disease control with the administration of adjuvant tyrosine kinase inhibitor (TKI) compared with adjuvant chemotherapy.^11^-^12^ Based on the evidence, we made a hypothesis that PORT might not be beneficial in most patients with *EGFR*-mutant stage III-pN2 lung adenocarcinoma, except for those with extra risk factors for LRR.

We performed the retrospective study to investigate the risk factors of LRR in *EGFR*-mutant stage III-pN2 lung adenocarcinoma after complete surgical resection. A nomogram was developed to subgroup patients with high and low LRR risk. The study will provide important evidence on further investigation on PORT candidates. Omission of PORT might be recommended for patients with extremely low risk of LRR, and the potential of PORT should be only examined in those with higher chance of LRR in future studies.

## Patients and Methods

### Ethics, consent and permissions

The study was conducted according to the ethical standards of the Helsinki Declaration. It was reviewed and approved by the Ethics Committee of Sun Yat-sen University Cancer Center (YB2017-047). Since it was a retrospective and anonymous study, a waiver of authorization was required and granted.

### Study population

Consecutive patients who had lung surgery and were sent for the* EGFR* mutation test during the time-frame spanning from September 2001 to December 2016 at either of Sun Yat-sen University Cancer Center, Tianjin Medical University Cancer Institute and Hospital, Union Hospital Cancer Center and The First Affiliated Hospital of Guangzhou Medical University were retrospectively screened. The following criteria were met for study inclusion: 1) histologically confirmed lung adenocarcinoma with sensitizing *EGFR* mutation (exon 19 or 21); 2) regarded as resectable according to pre-operative work-ups, including chest and upper abdominal computed tomography (CT) scans, bone scan, brain magnetic resonance imaging (MRI) and bronchoscopy, and in some cases, positron emission tomography (PET)/CT scan, endobronchial ultrasound-guided transbronchial needle aspiration (EBUS-TBNA) or mediastinoscopy; 3) resected completely; and 4) pathologically diagnosed with N2 disease. Patients were excluded if they received induction therapy or PORT, or had a postoperative follow-up time of less than six months. The staging was based on AJCC/UICC 8^th^ staging criteria.^13^

### N sub-staging

N sub-staging was collected and examined as a potential risk factor of LRR. Pre-treatment work-ups and pathology reports were carefully reviewed to confirm the status of N2 nodes. They were further sub-staged as unforeseen N2 (IIIA1-2), minimal N2/single station at staging (IIIA3), and bulky and/or multilevel N2 at staging (IIIA4), according to the Robinson Classification.^14^ IIIA1 was not separated from IIIA2 because intraoperative mediastinal lymph node pathological staging was not routinely performed.

### EGFR genotyping

Paraffin-embedded, formalin-fixed tissues obtained from surgical samples were prepared for the extraction of genomic DNA. *EGFR* mutation was tested via the amplification-refractory mutation system (ARMS) or direct sequencing depending on the technique used in each center.

### Pathological examination

An experienced pathologist, one for each center, reviewed the slides with hematoxylin-eosin staining, immunohistochemical staining and elastic staining. Complete resection was defined as free resection margins proven microscopically. The numbers of examined nodes were counted to reflect the quality of both lymphadenectomy and pathological examination. A cut-off of 16 examined nodes was used for further analysis because our previous work suggested it as a prognostic factor of overall survival (OS).^15^ Extranodal extension was defined as the invasion of malignant cells into perinodal adipose tissue through the nodal capsule. Tumor extension beyond the elastic layer, into the lymph and vascular system, or into the space surrounding a nerve was considered as visceral pleural invasion, lymphovascular invasion or perineural invasion, respectively. Skip lymph node metastasis was defined as N2 involvement without positive N1 node.^16^

### Surgery

Before surgery, all patients with multilevel or/and bulky N2 but refused neoadjuvant chemotherapy were discussed by a multidisciplinary team (MDT). The patients went straight to surgery unless that the tumor was regarded primarily resectable. The surgical procedure was decided according to the size and location of disease, patients' pulmonary function, cardiac function, and other comorbidities. Ipsilateral station 1 nodes were routinely dissected. Ipsilateral mediastinal lymph node dissection was performed, including the dissection of stations 2R, 4R, and 7-9 for right lung cancer and 4L, 5, 6, and 7-9 for left lung cancer.

### Adjuvant therapies

The adjuvant administration of chemotherapy (at least four cycles) and TKIs (at least two months) were reviewed and documented. Adjuvant chemotherapy was routinely recommended for all patients, in the light of the current guidelines.^6^-^7^ Either of the following two-drug regimens was administered with recommended dose: pemetrexed+cisplatin/carboplatin, paclitaxel +carboplatin, docetaxel+cisplatin/nedaplatin, vinorelbine detartrate+cisplatin, or gemcitabine+cisplatin/nedaplatin. Some patients treated between 2012 and 2015 in Tianjin Medical University Cancer Institute and Hospital were enrolled in a phase 2 clinical trial (NCT01683174), where the participants were randomized for adjuvant erlotinib or vinorelbine plus cisplatin. For those refused or showed intolerance to adjuvant chemotherapy, adjuvant TKI was discussed by patients and physicians as a substitute. Either gefitinib, erlotinib, afatinib or icotinib was administered. In some cases, after standard adjuvant chemotherapy, a maintenance use of TKIs was decided if an agreement was reached by patients and physicians, after weighing pros and cons.

### Follow-up

Chest and upper abdominal CT, and brain MRI were performed 1-2 months after the end of treatment, every 3-6 months in the first two years, and every 6-12 months after that.^6^ Bone scan, PET/CT and biopsy were performed if necessary. Any relapse within the ipsilateral hemithorax (except for multiple recurrent lesions in the ipsilateral lung) or regional lymph nodes was regarded as a locoregional recurrence; relapse elsewhere was considered distant metastasis.^17^ Both ultimate local and distant disease progression were documented.

### Statistical methods

The time from surgery to first locoregional relapse, first distant metastasis, death or last follow-up were recorded. The cumulative incidence function was used to calculate the probability of LRR and distant metastasis (DM), where death was considered as a competing event.^18^ Disease-free survival (DFS) was defined as the time from surgery to the first recorded treatment failure or death. OS was defined as the time from surgery to death from any cause. DFS and OS were assessed by the Kaplan-Meier method. The associations between clinicopathological variables and the incidence of LRR were assessed by employing competing risk regression analysis.^18^ Factors with *P*<0.10 in univariable analyses were incorporated into the proportional subdistribution hazard model and evaluated in multivariate analyses. P-values <0.05 (two-sided) were regarded as statistically significant. Missing data were not included in the statistical analysis. Statistical tests were conducted using SPSS 22.0 and R 3.0.2.

### Nomogram

Variables achieving statistical significance in the multivariable analysis were used to formulate a competing risk nomogram by R 3.0.2.^19^ The nomogram was built to evaluate the chance of LRR and to select high-risk patients for intensive adjuvant therapies in future. A high recurrence risk was defined as a nomogram-predicted 2-year cumulative incidence of LRR exceeding 10%. PORT might be spared in those with a low recurrence risk, while intensive adjuvant therapies should be considered in high-risk patients in future. Internal validations were performed. A concordance index (c-index) value was calculated to measure discrimination performance, and a calibration curve was obtained by plotting the observed incidence against the nomogram-predicted probability via a bootstrap method with 1000 resamples.

## Results

**Fig 1:**
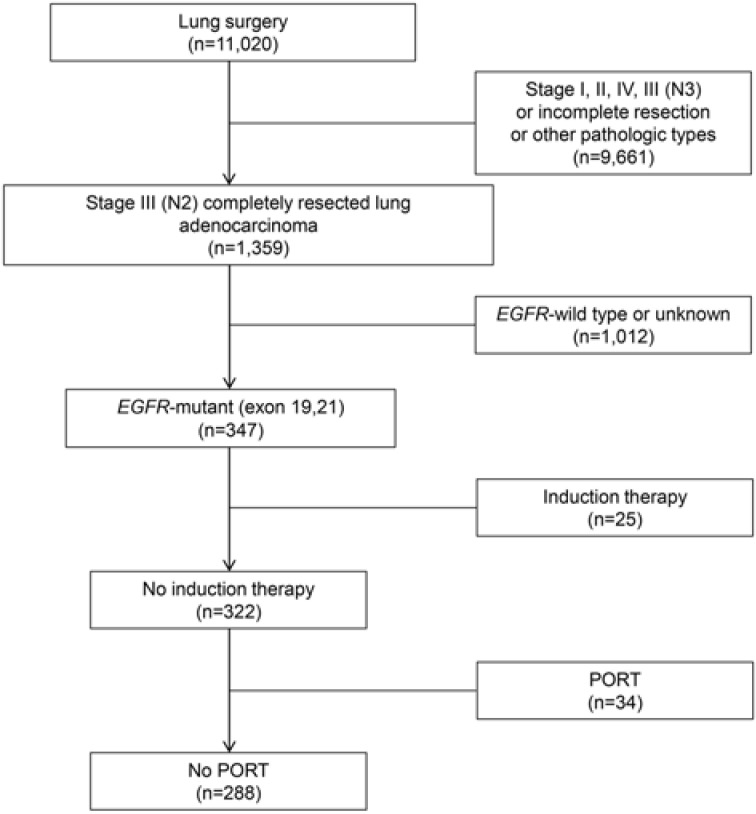
** Flowchart for patient enrollment.** *EGFR*: epidermal growth factor receptor; PORT: postoperative radiation therapy.

A total of 11,020 patients were screened. Among these, 288 consecutive cases met the study criteria and were included in the analysis (**Fig. 1**).** Table 1** details the clinicopathological characteristics and treatment-related parameters. There were 191 (66.3%) patients with IIIA1-2, 75 (26.0%) with IIIA3, and 22 (7.6%) with IIIA4. The 22 patients with IIIA4 did not receive adjuvant radiotherapy, due to contraindications (n=14), patient's refusal (n=3), economic reasons (n=3) or MDT recommendations (n=2). There were 152 (52.8%) patients diagnosed with exon 19 deletion, and 136 (47.2%) with exon 21 mutation, including 134 with L858R mutation and 2 with L861Q mutation. One hundred and ninety-four (67.4%) patients received adjuvant chemotherapy, while 67 (23.3%) received adjuvant TKIs. Of those had adjuvant TKIs, 20 were included in a clinical trial (NCT01683174), and 26 refused or were intolerant to adjuvant chemotherapy; the other 21 patients had adjuvant chemotherapy followed by adjuvant TKIs based on an individual decision made by the patients and physicians. The detailed regimens of adjuvant chemotherapy and TKIs are presented in **Table S1**.

### Patterns of recurrence

The median follow-up time was 28 (range: 6-133) months. Among the 288 patients, 46.5% (134/288) experienced relapse. Ultimately, 5.2% (15/288) had local recurrence only, 28.1% (81/288) had distant metastases only, 10.1% (29/288) had both, and 3.1% (9/288) had recurrence at unknown sites. The 2-year incidences of LRR and DM were 27.2% (CI, 16.3%-38.0%) and 58.2% (CI, 37.0%-79.3%), respectively. The sites of locoregional and distant relapses were detailed in **Table S2**.

After the first failure, 9 patients (6.7%, 9/134) had traditional Chinese medicine or palliative therapy because of economic reasons or personal decisions. The other 125 patients received salvage treatments including: TKI alone (n=72), chemotherapy alone (n=16), TKI+surgery (n=16), TKI+radiotherapy (n=15), chemotherapy+radiotherapy (n=4), and surgery alone (n=2).

### Risk factors and predictive nomogram for LRR

**Fig 2:**
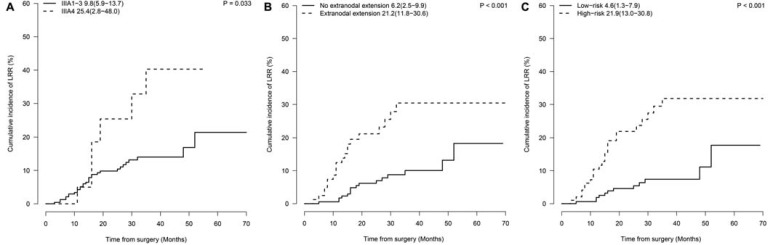
** Prognostic factors of locoregional recurrence.** Cumulative incidences of LRR by (A) N2 classification (2-year cumulative incidence of LRR, IIIA1-3 *vs* IIIA4: 9.8% *vs* 25.4%, *P*=0.033); (B) extranodal extension (2-year cumulative incidence of LRR, Yes *vs* No: 21.2% *vs* 6.2%, *P*<0.001); (C) risk-group (2-year cumulative incidence of LRR, low-risk *vs* high-risk group: 4.6% *vs* 21.9%, *P*<0.001). LRR: locoregional recurrence.

The N2 substage was associated with local recurrence, reflected by a 2-year incidence of LRR of 25.4% in IIIA4 patients and 9.8% in IIIA1-3 patients (*P*=0.033, **Fig. 2A**). Another risk factor for 2-year LRR rate was extranodal extension (21.2% *vs.* 6.2%, *P*<0.001,** Fig. 2B**). No statistically significant difference in LRR rates was observed between patients with or without adjuvant TKI treatment (2-year incidence of LRR, 7.9% *vs* 12.4%, *P*=0.22). In multivariate analysis, IIIA4 disease (hazard ratio [HR], 2.65; confidence interval [CI], 1.15-6.07; *P*=0.022) and extranodal extension (HR, 3.33; CI, 1.76-6.30; *P*<0.001) were demonstrated as independent risk factors (**Table 2**).

**Fig 3:**
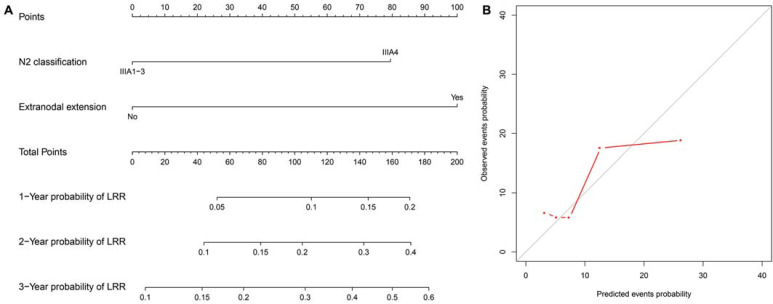
** LRR nomogram and calibration curves.** (A) The nomogram is created to estimate the 1-, 2- and 3-year cumulative incidence of LRR. The value of one individual patient is located at each variable axis. A straight line drawn upward determines the points received for each variable value. The sum of points is located on the total point axis, and then a straight line is drawn downward to the cumulative incidence of LRR axes to determine the 1-, 2-, or 3-year cumulative incidence of LRR. (B) The calibration curves predict the cumulative incidence of LRR at 1, 2 and 3 years. LRR: locoregional recurrence.

A nomogram predicting the 1-, 2- and 3-year cumulative incidences of LRR was created incorporating N2 status and pathological extranodal extension (**Fig. 3A**). The results of internal validation showed that the nomogram had a c-index of 0.715 (CI, 0.695-0.735) and was well calibrated for patients with low LRR risk (**Fig. 3B**). With a cut-off value of LRR risk of 10%, the patients were divided into the low-risk group (IIIA1-3 and no extranodal extension, 62.5%, 155/248) and the high-risk group (IIIA4 disease or/and extranodal extension, 37.5%, 93/248). The 2-year incidence of LRR was significantly different between the groups (low- vs high-risk groups, 4.6% *vs* 21.9%, *P*<0.001,** Fig. 2C**).

### Survivals

At the latest follow-up, 67 deaths were recorded among the whole cohort. The 2-year DFS and OS were 58.0% (CI, 51.7%-64.3%) and 89.7% (CI, 85.8%-93.6%), respectively. The estimated median DFS and OS were 28.0 (CI, 22.4-33.6) and 70.0 (CI, 61.0-79.0) months, respectively.

## Discussion

Our study investigated the patterns of recurrence and risk factors for LRR in *EGFR*-mutant stage III-pN2 lung adenocarcinoma after complete resection. Consecutive patients from four high-volume medical centers in China were combined. To the best of our knowledge, this was the first large retrospective study focusing on locoregional recurrence status in this specific subgroup.

In the current study, locoregional recurrence accounted for 15.3%, which was much less than that of distant metastasis (38.2%). The results were in good agreement with our previous study, in which local recurrence was 10.8% and 22% in *EGFR*-mutant and wild-type lung adenocarcinoma, respectively.^10^ Comparably, Mak et al.^20^ retrospectively examined locally advanced lung adenocarcinoma patients treated by chest radiotherapy. The rates of local recurrence and distant metastasis were reported 24% and 79% of *EGFR*-mutant patients, and in 46% and 66% of wild-type patients.

The presence of regional lymph nodes at initial presentation provides important information on the severity and behavior of stage III-pN2 lung adenocarcinoma. Robinson et al. 14 proposed four degrees of pre-treatment N2, demonstrating increasing aggressiveness of mediastinal nodes and difficulty in the radical resection. Distinct from wild-type patients, who had a higher chance (about 50%) to be diagnosed with IIIA4, the majority (66.3%) of *EGFR*-mutant patients had more unforeseen N2 (IIIA1-2).^21^ Unforeseen N2 reflects minimal mediastinal invasion and favorable survival.^22^,^23^ In this subgroup, the addition of adjuvant radiotherapy to chemotherapy did not provide extra benefit compared with chemotherapy alone.^24^ Minimal N2/single station was regarded as potentially resected cases proposed by the European Society for Medical Oncology (ESMO) Clinical Practice Guidelines.^7^ However, our data showed similar local control rates in the IIIA3 and IIIA1-2 subgroups, regardless of induction therapy. Less than 10% of patients presented bulky and/or multilevel nodes (IIIA4), which were related to a greater risk of incomplete resection,^7^ and a significantly higher risk of LRR and death was reported.^25^ For that reason, IIIA4 patients could have potential survival benefit from radiotherapy. Li et al.^26^ demonstrated a pathological response rate of 60% in bulky N2 disease treated by preoperative chemoradiation. Overall, since we analyzed consecutive data instead of performing matching, a tendency of unsuspected or resectable limited nodes in *EGFR*-mutant disease was uncovered. Baba T et al.^27^ also noted that positive nodes from adenocarcinoma were likely to be restricted in the low-risk node zone. This evidence might explain the favorable local control in our population, and the reason why only a small portion of patients received neoadjuvant therapy or PORT in clinical practice.

Extranodal extension could be recognized as a predictor of LRR in *EGFR*-mutant stage III-pN2 lung adenocarcinoma.^28^,^29^ Future studies assessing optimal radiotherapy should consider the stratification of capsule status. Although positive capsule is regarded as a potential indicator of PORT, further validation would be essential, because paradoxical results from another study suggested that PORT could only benefit those without extracapsular invasion.^30^ In recent studies, EBUS-TBNA showed an increased capacity in detecting the eventual presence of extracapsular extension,^31^,^32^ making it possible to investigate neoadjuvant radiotherapy in pre-surgically diagnosed extracapsular disease.

A nomogram predicting 1-, 2- and 3-year LRR rates was created, incorporating N2 status and pathological extranodal extension. We then divided patients into two groups with a cut-off value of the 2-year predicted LRR incidence of 10%. PORT might be spared in those without any of the risk factors (2-year incidence of LRR≤10%) to avoid radiation-induced injury and improve the quality of life.^33^ For those with pre-treatment bulky/multilevel N2 and/or extranodal extension (2-year incidence of LRR>10%), a propensity-score-matching study will be performed assessing the efficacy and toxicities of PORT. A peer report from Zhang Y et al. developed nomograms to predict the conditional risk of relapse in completely resected adenocarcinoma as well, including sex, age, tumor size, smoking history, tumor histology, visceral pleural invasion, lymphovascular invasion, and pathologic TNM stage as risk factors. The c-index was 0.743 predicting the overall risk of relapse. However, the study did not create a LRR risk nomogram for future investigation on intensive adjuvant local therapy.^34^

The current study had several limitations. First, 33.7% of patients, with initial suspected N2 proceeded directly to surgery instead of neoadjuvant treatments after the evaluation by thoracic surgeons.^35^,^36^ However, survival benefit could be found following surgery even in those patients with persistent single N2 involvement after induction therapy,^37^,^38^ leaving primary surgery as a possible choice. A second limitation was that, either ARMS or direct sequencing had been used because both of the techniques were accessible in different cancer centers. ARMS is believed to identify *EGFR* mutations more frequently, and those identified by ARMS tend to benefit more from TKIs,^39^ which might cause bias on estimating the effect of TKIs on LRR. Finally, as a retrospective study, selection bias and missing data were inevitable. Overall, the results should be validated in an external database and by randomized controlled trials.

## Conclusions

The low incidence of locoregional recurrence after complete resection reflects the distinctive nature of *EGFR*-mutant III-pN2 lung adenocarcinoma. Intensive adjuvant therapies such as PORT should only be considered in high-risk patients with pre-treatment bulky/multilevel N2 and/or pathological extra-nodal extension determined by the nomogram. Further study evaluating the optimal postoperative approach for completely resected *EGFR*-mutant N2-positive lung adenocarcinoma is warranted in the high-risk patients.

## Supplementary Material

Supplementary tables.

## Figures and Tables

**Table 1:** Patient characteristics

Characteristics	All patients
	n=288 (%)
Age	
≥60	126 (43.7)
<60	162 (56.3)
Sex	
Male	108 (37.5)
Female	180 (62.5)
KPS	
90-100	280 (97.2)
80	8 (2.8)
Pre-treatment T stage	
T1-2	270 (93.8)
T3-4	18 (6.2)
Pre-treatment N stage	
N0	88 (30.5)
N1	103 (35.8)
N2	97 (33.7)
Surgery	
Lobectomy	279 (96.9)
Pneumonectomy	9 (3.1)
*EGFR* mutation	
Exon 19	152 (52.8)
Exon 21	136 (47.2)
Smoking	
Yes	72 (25.0)
No	216 (75.0)
N2 classification	
IIIA1-3	266 (92.4)
IIIA4	22 (7.6)
Number of examined nodes	
<16	91 (32.4)
≥16	190 (67.6)
Missing data	7
Visceral pleural invasion	
Yes	94 (33.7)
No	185 (66.3)
Missing data	9
Lymphovascular invasion	
Yes	99 (39.3)
No	153 (60.7)
Missing data	36
Perineural invasion	
Yes	12 (5.3)
No	215 (94.7)
Missing data	61
Extranodal extension	
Yes	78 (31.5)
No	170 (68.5)
Missing data	40
Skip metastasis of lymph node	
Yes	80 (29.0)
No	196 (71.0)
Missing data	12
pT stage	
T1-2	260 (90.3)
T3-4	28 (9.7)
Adjuvant TKIs	
Yes	67 (23.3)
No	221 (76.7)
Adjuvant chemotherapy	
Yes	194 (67.4)
No	94 (32.6)

PORT: postoperative radiation therapy; KPS: Karnofsky performance score; *EGFR*: epidermal growth factor receptor; IIIA1-2: unforeseen N2; IIIA3: minimal/single station N2; IIIA4: bulky and/or multilevel N2; TKI: tyrosine kinase inhibitor. Missing data was presented but not included in analysis.

**Table 2:** Prognostic factors for LRR

	Univariable analysis	Multivariable analysis	
Variable	*P*	HR (95% CI)	*P*
Age (≥60 *vs* <60)	*0.64*		
Sex (male *vs* female)	*0.86*		
Pre-treatment T stage (T1-2 *vs* T3-4)	*0.78*		
Pre-treatment N stage (N0 *vs* N1 *vs* N2)	*0.22*		
Surgery (Lobectomy *vs* Pneumonectomy)	*0.55*		
*EGFR* mutation (exon 19 *vs* 21)	*0.064*	1.72 (0.85-3.57)	*0.13*
Smoking (yes *vs* no)	*0.92*		
N2 classification (IIIA4 *vs* IIIA1-3)	*0.033*	2.65 (1.15-6.07)	*0.022*
Number of examined nodes (<16 vs ≥16)	*0.68*		
Visceral pleural invasion (yes *vs* no)	*0.31*		
Lymphovascular invasion (yes *vs* no)	*0.66*		
Perineural invasion (yes *vs* no)	*0.95*		
Extranodal extension (yes *vs* no)	*<0.001*	3.33 (1.76-6.30)	*<0.001*
Skip metastasis of lymph node (yes *vs* no)	*0.27*		
pT stage (T1-2 *vs* T3-4)	*0.94*		
Adjuvant TKIs (Yes *vs* No)	*0.22*		
Adjuvant chemotherapy (Yes *vs* No)	*0.29*		

LRR: locoregional recurrence; HR: hazard ratio; CI: confidential interval; PORT: postoperative radiation therapy; *EGFR*: epidermal growth factor receptor; IIIA1-2: unforeseen N2; IIIA3: minimal/single station N2; IIIA4: bulky and/or multilevel N2; TKI: tyrosine kinase inhibitor.

## Abbreviations

ARMS: amplification-refractory mutation system
CI: confidential interval
c-index: concordance index
CT: computed tomography
DM: distant metastasis
DFS: disease-free survival
EBUS-TBNA: endobronchial ultrasound-guided transbronchial needle aspiration
*EGFR*: epidermal growth factor receptor
ESMO: European Society for Medical Oncology
HR: hazard ratio
IIIA1-2: unforeseen N2
IIIA3: minimal/single station N2
IIIA4: bulky and/or multilevel N2
KPS: Karnofsky performance score
LRR: locoregional recurrence
MRI: magnetic resonance imaging
OS: overall survival
PET: positron emission tomography
PORT: postoperative radiotherapy
TKI: tyrosine kinase inhibitor
