# Supplementary material for: Risk Prediction for Locoregional Recurrence in Epidermal Growth Factor Receptor-Mutant Stage III-pN2 Lung Adenocarcinoma after Complete Resection: A Multi-center Retrospective Study
Source: J Cancer. 2020 Aug 25;11(20):6114–21. doi: 10.7150/jca.47119 (PMC7477429; doi:10.7150/jca.47119)
Supplement: Supplementary file 1 — Supplementary tables. [file jcav11p6114s1.pdf]

Table S1 Adjuvant systemic treatments

Regimen	*N* (%)
Chemotherapy	203 (100)
Pemetrexed+cisplatin/carboplatin	157 (77.3)
Paclitaxel+carboplatin	8 (3.9)
Docetaxel+cisplatin/nedaplatin	8 (3.9)
Vinorelbine detartrate+cisplatin	24 (11.8)
Gemcitabine+cisplatin/nedaplatin	6 (3.0)
TKIs	70 (100)
Gefitinib	31 (44.3)
Erlotinib	20 (28.6)
Afatinib	2 (2.9)
Icotinib	17 (24.3)

Table S2 Relapse sites

Patterns of recurrence	*N* (%)
Locoregional relapse	47 (100)
Ipsilateral hemithorax	8 (17.0)
Regional lymph nodes	34 (72.3)
Unknown	5 (10.6)
Distant metastasis	116 (100)
Contralateral lung	22 (19.0)
Brain	22 (19.0)
Bone	16 (13.8)
Distant lymph nodes	5 (4.3)
Pleural cavity	4 (3.4)
Suprarenal gland	3 (2.6)
Liver	2 (1.8)
Multiple sites	36 (31.0)
Unknown	6 (5.2)
